# The Segmented Interview: Partitioning the Initial Free Recall Topics into Segments to Enhance Information Gathering and Lie Detection

**DOI:** 10.3390/bs15091163

**Published:** 2025-08-26

**Authors:** Haneen Deeb, Aldert Vrij, Mark Severino, Sharon Leal

**Affiliations:** 1 School of Psychology, Sport and Health Sciences, University of Portsmouth, Portsmouth PO1 2DY, UK; aldert.vrij@port.ac.uk (A.V.); sharon.leal@port.ac.uk (S.L.); 2 Los Angeles Police Department (Ret.), Los Angeles, CA 90012, USA

**Keywords:** segmented interview, lie detection, information gathering, investigative interview, structured interview, veracity cues, total details, complications

## Abstract

In standard investigative interviews, follow-up questioning from a free recall is typically based on the core topics of the free recall that are relevant to the event under investigation. We suggest the Segmented Interview as an alternative in which each free recall topic is partitioned into segments, and focused questioning occurs for each topic and segment separately, regardless of their relevance to the event under investigation. We expected the focused questioning of the Segmented Interview to elicit more details and Veracity cues than a Structured Interview. All participants (*N* = 80) completed three activities, of which only the second was different: Truth tellers visited a store, whereas lie tellers stole an envelope with money. Participants were then interviewed and provided a free recall, followed by open questions based on the Segmented or Structured Interview protocol. The Segmented Interview elicited more information and Veracity cues than the Structured Interview. These results suggest that the Segmented Interview may be a promising interview technique for eliciting information and detecting lies.

## 1. Introduction

The third author, Mark Severino, recalls an investigation of a robbery case that involved a female driver, her boyfriend, and two associates. The female said in her initial statement (free recall) that she waited in the car while her boyfriend and associates entered a vape shop to rob it and that they drove off to her boyfriend’s house after the robbery. Instead of asking about the robbery in general, Severino decided to partition the free recall to topics (how the robbery unfolded and driving to the boyfriend’s house) based on the female’s statement. He then divided each topic into segments and implemented focused questioning on each segment. Some of the segments of the topic ‘how the robbery unfolded’ included descriptions of (a) what the female did while waiting for her associates, (b) the boyfriend and associates, and (c) her feelings. When asked for descriptions of her boyfriend and associates, the female eventually admitted that her boyfriend and his associates were serial robbers who had previously robbed several pharmacies. What started as a single robbery ended up solving multiple robberies. The female’s initial statement was ten minutes long, but that one segment took thirty minutes to exhaust.

There is a tendency among investigative interviewers to ask questions about the target event under investigation rather than asking about every topic that is reported by the interviewee. A topic is a main activity occurring at a given location and time—as mentioned by the interviewee—which could have happened before, during, or after the event under investigation. An interviewer following a standard interview would not have necessarily asked for information on driving to the boyfriend’s house and may not have segmented the ‘how the robbery unfolded’ and asked about the boyfriend and associates if these are known to law enforcement. Without trying to know more about every aspect of the initial statement, information about previous robberies may not have been elicited.

In the present experiment, we suggest the so-called ‘Segmented Interview’ to examine the effects of objective segmentation and focused follow-up questioning on information gathering and lie detection. When using the Segmented Interview, the interviewer does not selectively partition the initial free recall into topics they deem to be relevant. Rather, the interviewer partitions all the free recall into topics. Also, instead of asking about each topic in a broad manner, the interviewer divides the topics into segments and asks open questions about each segment separately.

### 1.1. The Initial Free Recall and Follow-Up Questions for Gathering Information

An initial statement or free recall is the cornerstone of any investigative interview that focuses on information gathering (Powell & Snow, 2007). The United Kingdom’s PEACE interview is the most empirically tested interview model, and it is being widely implemented in different countries, including the United Kingdom (U.K.), Scandinavia, Australia, and New Zealand (Walsh et al., 2016). The most basic or standard interview within the PEACE model is the Structured Interview, in which the interviewee is first invited to provide a statement of what happened during the event in as many details as possible without being interrupted (Dando et al., 2009; Memon et al., 2010). However, not all information can be elicited in this free recall, even when interviewees are telling the truth (Fisher, 1995). While innocent interviewees are typically motivated to provide information that helps the interviewer resolve the case and prove their innocence (Granhag & Hartwig, 2008), it may be difficult for them to retrieve information about events that occurred a long time ago or that were emotionally charged (Geiselman & Fisher, 1985; Semmler et al., 2018). Also, innocent interviewees may believe that some information is insignificant, and they thus prefer not to waste the interviewer’s time with such information (Fisher et al., 2014). Unknown to interviewees, this information can be vital for resolving some cases (Brandon et al., 2018).

For Achieving Best Evidence (U.K. Ministry of Justice, 2022), it is recommended that investigative interviewers follow up on a free recall by using open and probing questions. Previous eyewitness research has shown that interviewees offer most details in a free recall, but asking follow-up questions can increase the number of new details by approximately 20% (Kontogianni et al., 2020). Follow-up questions enable interviewees to elaborate on the provided information and thus to clarify what they are trying to communicate and eliminate any perceived contradictions. Follow-up questions also encourage interviewees to provide more information that helps with resolving the case, show interviewees that the interviewer is interested in listening to them, and enhance opportunities for detecting deceit (Fisher & Geiselman, 1992; Hartwig et al., 2011).

The type of follow-up questions asked can be consequential in investigative interviews, and there is ample evidence that supports the use of open—rather than closed questions—for information gathering (Lamb et al., 2009; Oxburgh et al., 2010). For open questions to be effective, they should not be leading or suggestible (Meissner et al., 2014; Richardson et al., 1995) but solely based on the interviewee’s own (initial) statement and originally chosen words (witness-compatible questioning; Wells et al., 2006). Unlike closed questions (e.g., yes/no questions), which demand a response of very few words and are largely based on recognition memory, open questions demand a more elaborate response and are more accessible in memory, thus eliciting more detailed and accurate statements (Koriat & Goldsmith, 1994; Nunan et al., 2020).

While types of open questions have been defined and introduced differently in the memory and investigative interviewing literature (Oxburgh et al., 2010), these generally include Tell, Explain, Describe (TED) questions (e.g., ‘Tell me more about what happened; ‘Explain how you managed to escape’; ‘Describe what happened while you were at the shop’, and 5-WH (who, what, where, when, how) probing questions. The 5-WH cued-recall questions are more targeted than the TED questions (Caso et al., 2024; Griffiths & Milne, 2006). We therefore focus on TED questions in the present experiment.

The most frequently suggested open questions are the TED and 5-WH questions, but other distinctions can also be made. Powell and Snow (2007) classified open questions into depth and breadth questions. Depth questions are based on previously reported information (e.g., ‘You said you saw someone passing by, please describe that person’). Breadth questions request more information on a topic that was not disclosed previously (e.g., ‘What happened next?’). Depth questions often result in more (accurate) information than breadth questions (Danby & Sharman, 2023; Denne et al., 2024). We therefore only asked depth questions in the present experiment.

### 1.2. The Initial Free Recall and Follow-Up Questions for Detecting Lies

Lie tellers are less likely to provide details in their accounts than truth tellers (DePaulo et al., 2003). Lie tellers prefer to keep their accounts simple and to the point (Colwell et al., 2013; Hartwig et al., 2007). They choose to stay as close to the truth as possible so that they do not unintentionally contradict themselves or provide incriminating information (Leins et al., 2013; Strömwall & Willén, 2011). In contrast, truth tellers who report about a fully experienced event can provide a detailed statement as they generally want to report information that can help the interviewer resolve the case and prove their innocence (Granhag & Hartwig, 2015; Verschuere et al., 2021).

It is generally difficult to distinguish truth tellers and lie tellers based on free recall (Bond & DePaulo, 2006). Follow-up open questions urge interviewees to elaborate on their response, and the more someone talks, the higher the opportunities of lie detection (Hines et al., 2010). When follow-up probing (and closed) questions are asked, interviewees are obliged to provide more specific details on a topic. For lie tellers, that means providing a response they would not have otherwise provided, which may unveil their lie (Hartwig et al., 2011).

Researchers generally recommend the use of open questions to elicit Veracity cues (Evans et al., 2013). Asking open questions in a non-accusatory manner helps with building rapport and transferring the control of the interview to the interviewee (Geiselman, 2012). Making the interviewee comfortable in the interview should mitigate anxiety, which, in turn, leads to gathering more information and true confessions to a crime (Meissner et al., 2014).

### 1.3. Developing Interview Techniques That Enhance Information Gathering and Lie Detection

Given that the free recall by itself is not enough for gathering information and detecting lies, memory and lie detection researchers have developed novel interview techniques that enhance the elicitation of information and veracity cues. These techniques were based on or added to the Structured Interview. Some of these techniques involve mentally reinstating the context (Davis et al., 2005), sketching the event (Mac Giolla et al., 2017), providing information on a timeline (Hope et al., 2013), and clustering the event into categories (Paulo et al., 2017). To test the effectiveness of these interview techniques, researchers compare them to a free recall-only interview or to a Structured Interview (a free recall followed by open and sometimes closed questions; Geiselman & Fisher, 1985; Memon et al., 1997). The developed techniques have generally enhanced the recall of information and the elicitation of veracity cues relative to comparison groups. In the present experiment, we used the Structured Interview as the comparison group, in which a free recall was requested, followed by TED, depth questions (U.K. Ministry of Justice, 2022).

### 1.4. The Segmented Interview

We tested a novel interview technique—‘the Segmented Interview’—in which each topic in the initial free recall is partitioned into segments, and that is followed up by open-depth questions for every segment. For example, if the interviewee reports on what happened before, during, and after the event in a free recall, the interviewer can divide the interview into three topics: before, during (target event), and after the event. The interviewer then focuses on one topic at a time (regardless of its relevance to the event under investigation) and asks follow-up questions on each segment within a topic, thus exhausting all previously mentioned information in the free recall. The Segmented Interview aligns with practical recommendations that interviewers should not jump from one topic to another but focus on one topic at a time (Brandon et al., 2018; Fisher & Geiselman, 1992).

The Segmented Interview differs from a Structured Interview in at least four ways. First, in a Structured Interview, the topics chosen to probe are often based on the relevance of the topic to the event under investigation. They thus depend on the interviewer’s opinion of what topics may be relevant. The follow-up questioning phase also depends on whether the interviewer believes that a topic was adequately covered in the free recall. For example, it is stated in the Achieving Best Evidence guidance (U.K. Ministry of Justice, 2022, p. 84) that ‘Each topic not adequately covered in the witness’s (free recall) account should then be introduced’. This suggests that further questioning is not needed for some topics mentioned in the free recall if the interviewer believes they were adequately addressed. Thus, critical topics can go unnoticed if the interviewer decides they are irrelevant and/or have been adequately covered. With a Segmented Interview, follow-up questioning involves asking about every topic in the free recall, regardless of its relevance (refer to the opening example on descriptions of boyfriend and associates already known to the police). Thus, the questions are based on the totality of what was remembered in the free recall. If the interviewee mentions a topic, then it is sensible to assume the topic is of importance to the interviewee and of their perception of how the events unfolded, so it should be investigated.

Second, it is not clear in the Structured Interview how the free recall topics can be probed. While the Structured Interview protocol (U.K. Ministry of Justice, 2022) refers to the use of follow-up open and closed questions, it does not suggest segmenting the topics of the free recall, and it does not provide a structure on how to probe them. In the Segmented Interview, entire segments are devoted to each topic using focused questioning, and the interviewee concentrates on a single segment at a time. The interviewee can concentrate on specific incidents and time periods they would not have otherwise processed efficiently and reported (Leins et al., 2014; Paulo et al., 2017). This focused questioning slows the interview process for both the interviewee and interviewer and allows for a more in-depth retrieval of the event.

Third, while the Structured Interview demonstrates a good and evidence-based questioning technique, the Segmented Interview is expected to further facilitate memory recall and thus to enhance lie detection. The Segmented Interview should include more topics than the Structured Interview, and the topics are expected to include more segments and focused questions. Asking about each segment should facilitate the retrieval of other segments and perhaps of other topics. According to the spreading activation theory (Collins & Loftus, 1975), memory is a network that can be activated by memory cues; once one memory trace is activated by a cued-recall question, it follows that other memory traces are activated. As more information is disclosed, more segmentation and focused questioning can be implemented on the new information. With more information being reported, opportunities for detecting deception are also enhanced (Geiselman, 2012). 

Fourth, the Segmented Interview is expected to diminish resistance by interviewees more than the Structured Interview. In the example provided at the beginning of the paper, the female interviewee was initially resistant to giving information but shifted her strategy after the interview and topics were segmented. The focused nature of probing each segment may have urged her to provide more information to appear convincing and forthcoming. Interviewees may not expect that their free recall will be segmented, and unanticipated questions often elicit more information and veracity cues than anticipated questions (Vrij et al., 2009).

Lie tellers usually prepare responses to questions they anticipate (Chan & Bull, 2014). However, asking unanticipated questions should counter their preparation strategy and make it more difficult for them to respond as they try to avoid reporting incriminating information (Granhag & Hartwig, 2008). When asked unanticipated questions or when pressed for information, lie tellers may shift their strategies to ensure they appear cooperative (and honest), so they resolve this by (a) reporting non-incriminating information that comprise peripheral details irrelevant to the event under investigation (e.g., events happening before or after the target event; Alison et al., 2014), (b) providing scripted information based on general knowledge (e.g., common knowledge details; “employees were having lunch in the foyer at noon”; Vrij et al., 2019), and/or (c) in case they decide not to volunteer some information, using self-handicapping strategies to justify why they cannot provide the information (e.g., “It was dark, so I could not see what was happening”; Vrij et al., 2021).

The Segmented Interview and its unanticipated nature should not be problematic for truth tellers, as they do not usually use intentional strategies in interviews but only rely on their memory when answering questions (Johnson & Raye, 1981). In fact, segmenting the free recall should help truth tellers recall more information and explain away any information that came across as contradictory (Granhag & Hartwig, 2015). Truth tellers may thus become more able to provide core (central) details about the target event and complications, which are incidents that make an event more complicated (e.g., “I ordered a vegan pizza, but I received a vegetarian pizza”). Statements can typically be well understood without including complications, and truth tellers typically report them when they are encouraged to add more information. Lie tellers do not usually think of reporting complications, and reporting them goes against their inclination to keep stories simple (Hartwig et al., 2007; Maier et al., 2018).

In sum, the Segmented Interview is similar to the Structured Interview in that it is based on the notion that the initial free recall is rarely complete and that more information can be obtained by further probing relevant topics. However, unlike the Structured Interview, in the Segmented Interview, all topics mentioned in the free recall are included in the follow-up questioning phase, the topics are partitioned into segments, and focused questioning is applied for each segment separately. All these factors suggest that the Segmented Interview is more focused and is likely to enhance information gathering and lie detection beyond the Structured Interview.

In the present research, we compared the Segmented Interview and the Structured Interview on core and peripheral details, complications, common knowledge details, and self-handicapping strategies. The topics of the initial free recall were partitioned in the Segmented Interview but not in the Structured Interview. As Structured Interview protocols recommend that interviewees elaborate on relevant topics in the free recall (U.K. Ministry of Justice, 2022), one open follow-up question was asked on each topic in the Structured Interview condition. In both the Segmented and Structured Interviews, only open (TED) depth questions were asked about each topic, because (a) we wanted to standardise the questions and ensure that question type did not affect the findings, and (b) these are the most effective questions for eliciting reliable information (Caso et al., 2024; Danby & Sharman, 2023).

We tested a true/false alibi scenario (the alibi being a store visit). Participants engaged in activities that were relevant and irrelevant to the alibi (event under investigation). The rationale for these activities is that lie tellers often use embedded or omission lies so they include truthful aspects when they report an alibi (Leins et al., 2013). We thus wanted lie tellers to engage in non-incriminating activities (watching a video, checking if a lecturer was in her office) surrounding the mock crime (before and after stealing an envelope with money). That enabled us to examine (a) if lie tellers provided information about the non-incriminating activities (i.e., peripheral details) and (b) how the information lie tellers provided compared to that of truth tellers.

In line with previous research (Deeb et al., 2018; Vrij et al., 2021), we predicted that truth tellers will report more core details and more (core and peripheral) complications but fewer peripheral details, fewer (core and peripheral) common knowledge details, and fewer (core and peripheral) self-handicapping strategies than lie tellers (Hypothesis 1; Veracity main effect).

We also predicted that the focused questioning in the Segmented Interview would enable participants, particularly truth tellers, to elaborate on their initial free recall, which should, in turn, enhance opportunities for information gathering and lie detection (Geiselman, 2012). Thus, participants were expected to report more core and peripheral details, more complications, more common knowledge details, and more self-handicapping strategies in the Segmented Interview than in the Structured Interview (Hypothesis 2; Interview main effect). The differences between truth tellers and lie tellers in Hypothesis 1 were expected to be particularly pronounced in the Segmented Interview (Hypothesis 3; Veracity x Interview interaction effect).

The research and hypotheses were pre-registered on https://osf.io/xd6vu.

## 2. Materials and Methods

### 2.1. Participants

Participants were recruited at the University of Portsmouth through the participant pool, departmental databases, university portals, and social media platforms and received either 1.5 course credits or GBP 15 for taking part. The sample included 80 participants (M_Age_ = 22.04, SD_Age_ = 5.40) of whom 55 were females, 24 were males, and 1 was non-binary. The majority of participants were White (*n* = 41), followed by Black (*n* = 18), Asian (*n* = 17), and Arab (*n* = 1). Three participants were of mixed ethnicity. Participants were allocated randomly and equally (*n* = 20) to the four Veracity (lie teller, truth teller) and Interview (Segmented, Structured) conditions.

### 2.2. Procedure

#### 2.2.1. Truth Tellers

At the outset of the experiment, the experimenter gave truth tellers the keys to a locked room where they had to watch a 2.20-min documentary video about the city. After watching the video, they had to lock the room and return the keys to the experimenter. Next, the experimenter asked them to go to a store near the department to search for a mango-based hand cream, as a university scientist is interested in testing that item but wants to know first if the item is still being sold in the store. Participants were instructed to take a photo of the item and to show it to the experimenter as evidence that it is being sold in the store. After they returned to the department, the experimenter asked participants to check if a lecturer was in her office.

Once truth tellers completed the three activities and returned to the experimenter’s room, they were met by the interviewer who stated: “An envelope with money disappeared from room […], and everyone currently in the department will be interviewed. I will thus be interviewing you in a few minutes”. The interviewer then left the room and returned after three minutes to take the participant to the interview room.

After the interviewer left the room, the experimenter provided the following motivation instructions: “As you saw, the interviewer will interview you about your whereabouts. Please be truthful and say that you have been to the store. So the store will be your alibi. If you convince the interviewer that you are honest, you will receive the reward of £15/the course credits. If you are not convincing, you will not receive the reward. So please convince the interviewer that you were at the store. You can prepare for the interview until the interviewer is back”. In reality, all participants (including lie tellers) received the reward.

#### 2.2.2. Lie Tellers

Lie tellers underwent a similar procedure. Their first activity was to watch the video. The second activity, however, was not to go to the store but to steal an envelope with money from a room without anyone noticing. After they completed the second activity, the experimenter took the envelope from them and asked them to do the final activity, which was to check if the lecturer was in her office.

After they returned to the experimenter, they were met by the interviewer, who informed them they would be interviewed (same instructions as for truth tellers). When the interviewer left, the experimenter instructed the lie tellers as follows: “As you saw, the interviewer will interview you about your whereabouts. I need you to convince the interviewer that you have not taken the envelope, or else you and I will be implicated. Thus, you have to lie to the interviewer and say that you have been to a nearby store during that time. So the store will be your alibi. If you convince the interviewer that you are honest, you will receive the reward of £15/the course credits. If you are not convincing, you will not receive the reward. So please convince the interviewer that you were at the store. You can prepare for the interview until the interviewer is back.”

#### 2.2.3. The Interview

One of two research assistants, blind to participants’ Veracity conditions, interviewed participants. The interview protocol (including questions, examples of follow-up questions, and guidance to interviewers) can be found in the Supplementary Materials. For all participants, the interview started with an invitation for a free recall: “It came to my attention that an envelope with money went missing during the past half an hour or so. I am thus interviewing all people present within the department. Please tell me about your whereabouts over the past half an hour or so.”

After participants provided the initial free recall, the interviewer stated, “I will be asking you for more information to clarify what you previously mentioned.” The interviewer then proceeded to ask the follow-up questions, which they formulated based on the Interview condition (Segmented vs. Structured) and based on what the participant mentioned in the free recall. The interviewer always used the participant’s own words (from the free recall) to formulate the questions. The wording of the Structured and Segmented Interview questions was always the same as in the examples below (only the topic changed). The order of the questions (on activities) was based on how the information was remembered by the participant (Brandon et al., 2018; Fisher & Geiselman, 1992).

Participants in the Structured Interview condition were asked one open question about each main activity (topic) they reported. Thus, a participant who has reported about all three activities will be asked the following questions:

1. You mentioned going to a room to watch a video. Tell me everything you can remember around that time.
2. You mentioned going to a store to look for an item. Tell me everything you can remember around that time.
3. You mentioned going to check if a lecturer was in her office. Tell me everything you can remember around that time.

Fewer or more questions could be asked, depending on the topics (activities) mentioned by the participant in the free recall. The ‘watching video’ and ‘checking the lecturer was in her office’ activities are not critical to the event under investigation (the alibi). We would expect a practitioner not to ask follow-up questions on these peripheral activities in a Structured Interview. However, we included questions about these activities to standardise the experimental procedure and to make the questions as comparable as possible between the Structured and Segmented Interviews.

For the Segmented Interview, the initial free recall was partitioned into topics, and the topics were then partitioned into four segments. One open question was asked about each segment. In the examples below, (1), (2), and (3) are topics, and (a), (b), (c), and (d) are segments. As in the Structured Interview, the (number of) topics queried depended on the topics mentioned by the participant in the free recall.

1. You mentioned going to a room to watch a video.
  - Tell me everything you can remember on the way to the room.
  - Tell me everything you can remember about what you did while in the room.
  - Tell me everything you can remember about the room.
  - Tell me everything you can remember on the way from the room.
2. You mentioned going to a store to look for an item.
  - Tell me everything you can remember on the way to the store.
  - Tell me everything you can remember about what you did while at the store.
  - Tell me everything you can remember about the store.
  - Tell me everything you can remember on the way from the store.
3. You mentioned checking if a lecturer was in her office.
  - Tell me everything you can remember on the way to the lecturer’s office.
  - Tell me everything you can remember about what you did while next to the lecturer’s office.
  - Tell me everything you can remember about the lecturer’s office.
  - Tell me everything you can remember on the way from the lecturer’s office.

#### 2.2.4. The Post-Interview Questionnaire

After the interview had finished, participants were asked to complete an online questionnaire created via Gorilla. They first rated on 7-point scales (1 = disagree to 7 = agree) the extent to which they were truthful and motivated, their perceptions of the interviewer and the interview questions, and their familiarity with their alibi (see Table 1 for the exact questions). They also provided their demographics and reported the convincing strategy or strategies they used to appear convincing during the interview (open question).

### 2.3. Coding

The interviews were transcribed verbatim and coded for the number of core and peripheral details, for which we used the PLATO coding scheme (see Deeb et al., 2022). PLATO stands for person, location, action, time, and object details. Person details involve the mention and physical descriptions of persons (“There was an employee, a male that helped me. He had a shaved head and a ponytail, and he was wearing pink” includes six person details). Location details refer to directions and static places and their descriptions (“When leaving the psychology building, I saw my friend who was just off to the lab. I then walked to Guildhall square, underneath the Civic offices, past the station, until I reached the fountain” includes nine location details). Action details are action verbs (“I got a key, entered the room, and watched the video” includes three action details). Temporal details denote time (“It was a two-minute video clip on the city. After watching it, I left the room” includes three temporal details). Object details refer to non-static objects and their descriptions (“I was looking for the mango hand cream, but they only had shea butter and blossom cherry” includes five object details).

Core and peripheral details were distinguished. Core details referred to the alibi, so everything reported about the walk to/from the store and being in the store was coded as core details. Peripheral details were irrelevant details that happened before or after the alibi (before leaving the department for the store and after returning to the department from the store). Thus, a participant who has only spoken about the store would have reported only core details and no peripheral details. Participants who have mentioned the store visit and watching the video, for example, would have reported core and peripheral details.

We also coded the interviews for (a) complications, which are information that make a narrative more complicated than necessary (“I could not find the hand cream so I had to ask a member of staff”), (b) common knowledge details which involve scripted information that is commonly known (“The floor—it was pretty generic. It’s kind of offices and staff there”), and (c) self-handicapping strategies which are justification for not being able to provide information (“I had my headphones on so I was not really focusing on what was happening”). Complications, common knowledge details, and self-handicapping strategies were then marked as core or peripheral.

In the free recall, all core and peripheral (PLATO) details, complications, common knowledge details, and self-handicapping strategies were coded. In the follow-up responses, only new core and peripheral (PLATO) details, complications, common knowledge details, and self-handicapping strategies were coded.

The first author coded all the transcripts for all variables. To assess inter-rater reliability, the second author coded all the transcripts for complications, and a research assistant coded 42 (53%) random transcripts for core and peripheral (PLATO) details, common knowledge details, and self-handicapping strategies. The first author offered training sessions to the research assistant. After the coding scheme was explained, the research assistant attempted to code one transcript, for which she received feedback. This was followed by further attempts and feedback until she was able to code the transcripts independently.

Inter-rater reliability was measured using the Intra-Class Correlation (ICC) coefficient (single-measure scores). Inter-rater reliability is poor for ICC values less than 0.40, fair for values between 0.40 and 0.59, good for values between 0.60 and 0.74, and excellent for values between 0.75 and 1 (Hallgren, 2012). Inter-rater reliability was excellent for core (PLATO) details (ICC = 0.96, 95% CI [0.92, 0.98]), peripheral (PLATO) details (ICC = 0.93, 95% CI [0.87, 0.96]), and complications (ICC = 0.76, 95% CI [0.65, 0.84]). For the analyses, we used the complications scores by the second author, as he is more experienced in this coding scheme.

The number of self-handicapping strategies was very low (the first author found only five self-handicapping strategies across all transcripts), so inter-rater reliability could not be calculated, and this variable was not analysed for hypothesis testing. The number of common knowledge details was also low (the first author detected 26 common knowledge details across all follow-up responses and only 2 in the free recall). Reliability was good (ICC = 0.66, 95% CI [0.45, 0.80]) for common knowledge details in the follow-up responses, but we could not analyse this variable due to its low frequency count.

Participants’ self-reported strategies to appear convincing (in the post-interview questionnaire) were also coded. The first author categorised participants’ responses into themes, such that similar responses were grouped together under a single theme. When the same response could fit in more than one theme, it was allocated to each of those themes. To measure inter-rater reliability, a research assistant coded all the responses based on the themes generated by the first author after receiving definitions and examples of each theme. Inter-rater reliability agreement was substantial, Cohen’s κ = 0.77.

## 3. Results

We carried out generalised linear models (GLMs) for all analyses with Veracity (lie teller, truth teller) and Interview (Segmented, Structured) as factors. The use of generalised linear models deviates from the pre-registration, but that was decided following peer review, because these analyses are more robust than multivariate analyses of variance and allow for a more comprehensive interpretation of the variables. We performed the analyses for a negative binomial distribution (Winter & Bürkner, 2021). To enhance the interpretation of the findings, we also conducted Bayesian analyses to test the likelihood of the data under the null hypothesis (H0) and the alternative hypothesis (H1). We compared four models: two models with the individual factors (Veracity; Interview), one model that combined the two factors, and the null model. We used the default JZS *r*-scale of 0.354 as prior and 0.25 as uniform prior odds. We report BF_10_ scores. BF_10_ scores above 3 suggest support for H1 over H0, and BF_10_ scores close to 1 indicate that no evidence can be derived from the data for either hypothesis (Jarosz & Wiley, 2014). We used JAMOVI 2.13.18 statistical software for the analyses.

### 3.1. Number of Follow-Up Core and Peripheral Topics

We divided the topics mentioned in the free recall into core topics and peripheral topics. Participants reported an average of 1.01 core topics (SD = 0.11, Min = 1, Max = 2) and 0.70 peripheral topics (SD = 0.77, Min = 0, Max = 2). A GLM with the number of core topics as the dependent variable did not reveal significant differences for Veracity or Interview, $\chi^{2}$ (1) = 0.01, *p* = 0.913. The GLM with the number of peripheral topics as the dependent variable revealed a significant effect of Veracity, $\chi^{2}$ (1) = 5.34, *p* = 0.021, exp(B) = 1.91, 95% CI [1.10. 3.43]. Truth tellers, M =0.93, SD = 0.80, 95% CI [0.67, 1.18], reported more peripheral topics than lie tellers, M = 0.48, SD = 0.68, 95% CI [0.26, 0.69]. The Interview effect was not significant, $\chi^{2}$ (1) = 2.04, *p* = 0.153, exp(B) = 1.50, 95% CI [0.86, 2.68]. A similar number of peripheral topics was reported in the Segmented Interview, M = 0.85, SD = 0.80, 95% CI [0.59, 1.11], and in the Structured Interview, M = 0.55, SD = 0.71, 95% CI [0.32, 0.78].

### 3.2. Number of Follow-Up Questions

Participants were asked an average of 4.53 follow-up questions (SD = 3.76, Min = 1, Max = 12). The GLM showed a significant effect of Interview, $\chi^{2}$ (1) = 166.42, *p* < 0.001, exp(B) = 4.81, 95% CI [3.68, 6.39]. The average number of follow-up questions in the Segmented Interview, M = 7.50, SD = 3.16, 95% CI [6.49, 8.51], was significantly higher than that in the Structured Interview, M = 1.55, SD = 0.71, 95% CI [1.32, 1.78]. The Veracity effect was not significant, $\chi^{2}$ (1) = 3.06, *p* = 0.080, exp(B) = 1.28, 95% CI [0.97, 1.69]. Truth tellers, M = 5.15, SD = 4.11, 95% CI [3.84, 6.46], and lie tellers, M = 3.90, SD = 3.32, 95% CI [2.84, 4.96], were asked a similar number of follow-up questions.

### 3.3. Interview Duration

The average interview duration was 4 min 2 s (SD = 2.42, Min = 1.00, Max = 11.28). A GLM with interview duration as the dependent variable showed a significant effect of Interview, $\chi^{2}$ (1) = 52.00, *p* < 0.001, exp(B) = 2.44, 95% CI [1.90, 3.17]. The Segmented Interview (free recall and follow-up questions combined), M = 5 min 13 s, SD = 2.38, 95% CI [4.37, 5.89], Min = 1.57, Max = 11.28, was significantly longer than the Structured Interview (free recall and follow-up questions combined), M = 2 min 10 s, SD = 1.22, 95% CI [1.71, 2.49], Min = 1.00, Max = 7.00. The Veracity effect was not significant, $\chi^{2}$ (1) = 2.12, *p* = 0.145, exp(B) = 1.21, 95% CI [0.94, 1.56]. The interviews of truth tellers (M = 4 min 35 s, SD = 2.35, 95% CI [3.20, 4.71]) lasted as long as those of lie tellers (M = 3 min 28 s, SD = 2.47, 95% CI [2.49, 4.07]).

### 3.4. Post-Interview Questionnaire

We ran GLMs (with Veracity and Interview as factors) on all 14 items (see Table 1 and Table 2) in the post-interview questionnaire rated on 7-point scales (1 = disagree to 7 = agree). The significant Veracity effects are shown in Table 1, and the significant Interview effects are reported in Table 2. Truth tellers were significantly more likely than lie tellers to be truthful, to think that the interviewer believed them, and to report details they perceived as important. Truth tellers were marginally more likely to expect to be asked fewer questions, but this was not supported by the Bayesian analysis. The Bayesian analysis also showed that truth tellers seem to have been more motivated than lie tellers. However, motivation, ratings of the interviewer, and familiarity averages were high among both truth tellers and lie tellers. Both Veracity groups thought that the interview was not very difficult and expected to be asked more questions during the interview.

Compared to participants in the Structured Interview, participants in the Segmented Interview were significantly more likely to believe the interview was cognitively demanding and to expect to be asked fewer questions. In both Interview conditions, motivation and perceptions of the interviewer were high, and the interview was rated as not difficult.

An interaction effect emerged for cognitive load, $\chi^{2}$ (1) = 3.96, *p* = 0.047, exp(B) = 0.92, 95% CI [0.38, 0.99]. Simple effects revealed that lie tellers perceived the Segmented Interview, M = 4.40, SD = 1.76, 95% CI [3.58, 5.22], to be significantly more cognitively demanding than the Structured Interview, M = 2.30, SD = 1.75, 95% CI [1.48, 3.12]. Truth tellers perceived the Segmented Interview, M = 4.00, SD = 1.86, 95% CI [3.13, 4.87], and the Structured Interview, M = 3.40, SD = 2.09, 95% CI [2.42, 4.38], to be similarly demanding.

Participants answered an open question on the convincing strategy or strategies they used during the interview to appear convincing. The self-reported strategies appear in Table 3. Truth tellers were more likely than lie tellers to report providing a detailed (“I explained my whereabouts in detail”) and honest account (“I was telling the truth”), adding details about their surroundings (“I mentioned all of the areas I passed through”), including significant details (“I tried to remember key things such as where the product was”), and acting in a friendly manner (“I used light humour”). Lie tellers were more likely than truth tellers to self-report using an embedded lie (“I based it off on where I have been previously today”) and providing a simple account (“not overcomplicating anything to not get caught in the lie”).

### 3.5. Hypotheses Testing

We calculated the total core and peripheral details scores. For the core details score, we summed the core PLATO details in the free recall + new core PLATO details in the follow-up responses. Thus, the formula of the core details score = (core person details + core location details + core action details + core temporal details + core object details in the free recall) + (new core person details + new core location details + new core action details + new core temporal details + new core object details in the follow-up responses). To calculate the peripheral details, we summed the peripheral PLATO details in the free recall + new peripheral PLATO details in the follow-up responses.

We also calculated the core and peripheral complications scores. For the core complications score, we summed core complications in the free recall and new core complications in the follow-up responses. The formula of the peripheral complications score = peripheral complications in the free recall + new peripheral complications in the follow-up responses.

We ran a GLM with Veracity (lie teller, truth teller) and Interview (Segmented, Structured) as factors and each of the core details, peripheral details, core complications, and peripheral complications as dependent variables. Significant Veracity effects emerged for peripheral details, core complications, and peripheral complications, but not for core details. Truth tellers provided more peripheral details and complications than lie tellers (see Table 4). However, the Bayesian analysis did not support the results for peripheral details. As for the Interview condition, significant effects emerged for all variables, with the Segmented Interview eliciting more detail types than the Structured Interview, and the Bayesian analyses supported these results (see Table 5).

We followed up the analyses with simple effects. As Table 6 shows, truth tellers provided more core complications and peripheral complications in the Segmented Interview and in the Structured Interview. However, the Bayesian analyses supported these results for the Segmented Interview only, with very strong evidence emerging for core complications.

### 3.6. Proportion of New Information

To understand the extent to which new information was elicited in the follow-up questions in each Interview condition, we calculated the means of core and peripheral details in the free recall and in the follow-up responses. As shown in Table 7, the (new) core and peripheral details means for the Structured Interview condition are almost similar in the free recall and in the follow-up responses (accounting for 50–54% new details). However, the Segmented Interview resulted in at least three times more core and peripheral details in the follow-up responses; new core details comprised 79% of the overall core details, and new peripheral details comprised 74% of the overall peripheral details.

## 4. Discussion

### 4.1. The Segmented Interview and Information Gathering

In line with our predictions, the Segmented Interview resulted in more information being reported than the Structured Interview. This was true for core details, peripheral details, core complications, and peripheral complications, which demonstrates that the Segmented Interview—at least when open-depth questions are asked—is not limited to eliciting information on the event under investigation but may also aid the interviewer in collecting information about various topics. The results also show that reporting about alibi-irrelevant details in the Segmented Interview does not negatively affect the elicitation of alibi-relevant (core) details.

New core and peripheral details comprised 74–79% of the overall core and peripheral details in the Segmented Interview, but only 50% in the Structured Interview. These proportions are high and mean that participants did not provide much information in the free recall. This finding aligns with previous lie detection research (Tekin et al., 2015; van Beek et al., 2022). Interviewees have more control over the free recall than over follow-up questions, as they can report as much information as they will in the free recall (Hartwig et al., 2011). Thus, they may report minimal or key information (about one or more activities) in the initial recall and then decide on how to respond as more questions are asked. For truth tellers, this would have meant reporting information they were confident about to ensure the accuracy of their free recall (Goldsmith et al., 2002). For lie tellers, that meant providing vague and evasive free recalls.

Indeed, in the post-interview questionnaire, lie tellers self-reported providing simple and less detailed statements, whereas truth tellers were more likely to report including information that was perceived as important. Also, all participants in the Structured Interview—and less so in the Segmented Interview—expected more questions to be asked. That in turn explains why participants, in general, did not find the interviews to be difficult, although those in the Segmented Interview condition believed that the interview required more cognitive effort and expected to be asked fewer questions.

More questions were asked in the Segmented Interview than in the Structured Interview. By segmenting the topics, the interviewer necessarily asks more questions. However, the number of questions alone does not necessarily determine the richness of information elicited; instead, it is the quality of questions that yields this effect (Dando et al., 2009; Russano et al., 2024). It is thus more likely that what resulted in greater information yield in the Segmented Interview is the segmentation and the use of more focused, in-depth (open and appropriate) questions that exhausted the narrative. We expect interviewers to segment the topics even further in real time as more information is provided, which by default means that more focused depth questions will be asked. While interviewers employing the Structured Interview will also ask more questions, they do not necessarily exhaust every topic as in the Segmented Interview.

### 4.2. The Segmented Interview and Lie Detection

Differences between truth tellers and lie tellers emerged for complications, but not for (PLATO) details. We expected truth tellers to report more details, particularly core details, than lie tellers in line with previous research (DePaulo et al., 2003; Leal et al., 2018). While the core details’ means in Table 4 were higher for truth tellers than for lie tellers, lie tellers’ scores were also high. Perhaps in our alibi scenario, lie tellers tried to provide as much core information as possible so that their story appears believable. This should have been easy in the current scenario, as lie tellers self-reported in the post-interview questionnaire that they were familiar with the alibi.

The results for peripheral details were not expected. The Bayesian analyses showed weak support for our hypothesis, and the means were in the opposite direction to that predicted, with truth tellers reporting more peripheral details than lie tellers. Truth tellers reported more peripheral topics than lie tellers, so it follows that they were asked more peripheral follow-up questions and thus reported more peripheral details. It is likely that truth tellers reported the peripheral activities because they wanted to be cooperative and to offer a complete picture of all activities they completed prior to the interview—thus providing detailed statements on the alibi-relevant and alibi-irrelevant activities—even though they were only instructed to talk about the alibi. Conversely, lie tellers may have wanted to maintain simplicity and report alibi-relevant details only.

Truth tellers reported more core and peripheral complications than lie tellers. Complications have received strong empirical evidence as a veracity cue, especially when compared to total details (Vrij et al., 2021). The odds ratios of the complications effects were large, and the Bayesian analyses provided strong support for these results. This is advantageous in real life as complications are easier to code in real time by interviewers (Vrij et al., 2023) compared to total details, given that complications represent units of information (multiple details) rather than a single unit of information (single detail).

Simple effects demonstrated that core and peripheral complications were the only cues that significantly differentiated truth tellers and lie tellers in the Segmented and Structured Interviews. However, the Bayesian analyses supported the alternative hypothesis for the Segmented Interview only, and the results were particularly pronounced for core complications. That reiterates that complications—particularly when looking at the core story—are strong veracity cues, and they can become more evident when the Segmented Interview is employed.

### 4.3. Practical Significance of the Segmented Interview

The interviewer applying the Segmented Interview follows rapport-building and evidence-based protocols, refrains from being judgmental, avoids biases, and bases the follow-up questions and their wording on the interviewee’s statement (Gabbert et al., 2021; Wells et al., 2006). The interviewer is not limited by the questions they can ask. For example, asking questions about the interviewee’s beliefs and opinions (evocation) on specific segments can be more useful than one would expect.

It is generally difficult to standardise the follow-up questioning phase because the questions are usually formulated based on the initial free recall. However, we tried to standardise the Segmented Interview by asking only open-depth questions and by limiting the number of questions to four for each topic (one question per segment). Given that the Segmented Interview elicited substantially more information than the Structured Interview and that the effect sizes were large, we expect real-life Segmented Interviews to elicit even more details as more questions are typically asked based on the free recall and also based on new information reported. Furthermore, the interviewer does not necessarily have to ask only depth questions. Breadth questions can also be useful because they can broaden the scope of the narrative and can elicit information about topics not initially mentioned by the interviewee in the free recall. 

We believe that the Segmented Interview may be used in combination with other interview techniques such as category clustering recall (Paulo et al., 2017) and the strategic disclosure of evidence where evidence exists (Granhag et al., 2013) to enhance information gathering and lie detection. The techniques used may vary depending on the segment. If the interviewee is resistant in a specific segment and does not provide sufficient information, the Model Statement interview technique (Leal et al., 2018) can be used to encourage reporting of more information. In another segment where the interviewer is keen on knowing if the interviewee is lying or telling the truth, they can ask unanticipated questions about temporal and spatial details. Moreover, a single timeline (Hope et al., 2013) can be created for the whole interview, such that the interviewee adds information to the timeline as each segment is exhausted. In the example at the beginning of the article, the female used a sketch plan in one of the segments to describe (draw) where each person was seated in the vehicle. The sketch also revealed conversations she heard between the boyfriend and associates.

### 4.4. Limitations and Future Research Directions

We recruited 80 participants based on the pre-registered power analysis for a multivariate analysis of variance. While we found Veracity differences between Interview conditions, our study may have been underpowered to detect more profound differences. Running the study with a larger sample size may have enhanced these effects.

The interview was short, whereas real-life interviews are typically longer and can happen over several days (Soufan, 2011). Probing a segment can take time, as mentioned in the robbery example in the Introduction. In the lab, it is difficult and sometimes unethical to run long interviews or make participants do as many activities as a criminal would. Nonetheless, we tried to add as many activities as possible so that participants can have enough activities to talk about and to increase sensitivity. We expect the effects to be larger in real-life interviews.

We asked only one follow-up question per topic in the Structured Interview and one follow-up question per segment in the Segmented Interview. A real-life interview is likely to include more follow-up questions on each topic and segment. For example, the segments in the Segmented Interview can be partitioned even further (e.g., ‘Describe the street layout’, ‘Tell me more about the staff in the store’). Also, the interviewer can ask more questions in real time as the interviewee reports new information. We decided against asking further questions because that would make the interviews very long, and the questions would vary within the same interview condition, which would affect the standardisation of protocols. We expect the differences between the interview conditions to be more pronounced in real life, where more focused questioning occurs for the Segmented Interview than for the Structured Interview.

Self-handicapping strategies and common knowledge details did not occur frequently enough to examine them. This is likely to happen when a context does not allow for the elicitation of specific cues (Ewens et al., 2015; Nahari et al., 2019). Perhaps the nature of the activities and the timing and length of the interview were not suitable for eliciting these cues. For example, a common self-handicapping strategy offered by interviewees is “I cannot remember”. Participants could not use this self-handicapping strategy in the present experiment as the interview occurred immediately after the event. Relatedly, it is important to examine the Segmented Interview in other contexts than alibi settings, such as intelligence and immigration settings, where eliciting information is vital.

Inaccurate details were not examined due to the nature of the activities, as we could not establish ground truth. While follow-up questions may have resulted in new information, they could have risked reduced accuracy. This may be especially true for truth tellers when reporting peripheral details (Herlihy et al., 2012). We preferred that participants engage in real experiences rather than watch a video stimulus (in which ground truth can be established) to mirror real-life experiences and enable segmentation. In future research, participants’ activities can be tracked to establish ground truth and thus measure (in)accurate details.

The interview occurred immediately after the target event. In real life, there is usually a delay between the interview and the event. The longer the delay, the more likely the interviewees will forget information and will provide inaccurate details (Murre & Dros, 2015). The effects of delay on the effectiveness of the Segmented Interview need to be considered.

## 5. Conclusions

We have shown that the Segmented Interview is effective for gathering information beyond a Structured Interview. The Segmented Interview serves as a mnemonic by segmenting and exhausting all free recall topics and by using focused open-depth questions for each segment. The Segmented Interview also seems to increase differences between truth tellers and lie tellers, particularly when looking at (core) complications. More research is needed to corroborate these results in other contexts and with respect to the accuracy of core and peripheral information provided. To conclude—and in the third author’s opinion—obtaining as detailed a lie (or a truth) as possible is better than obtaining a weak confession.

## Figures and Tables

**Table 1:** Descriptive and inferential statistics for the 7-point scale ratings in the post-interview questionnaire as a function of veracity.

Questionnaire Item	Truth Tellers		Lie Tellers		$\chi^{2}$ (1)	*p*	exp(B) [95% CI]	BF_10_
	M [SD]	95% CI	M [SD]	95% CI				
I was truthful during the interview.	6.90 [0.30]	6.80, 7.00	1.83 [1.06]	1.49, 2.16	126.22	<0.001	3.80 [2.95, 4.96]	8.34 × 10^39^
I was motivated to be believed by the interviewer.	6.48 [1.24]	6.08, 6.87	5.43 [1.80]	4.85, 6.00	3.74	0.053	1.20 [1.00, 1.43]	11.41
The interviewer believed me.	5.55 [0.93]	5.25, 5.85	4.33 [1.33]	3.90, 4.75	6.15	0.013	1.29 [1.05, 1.57]	2039.47
The interviewer was listening carefully to me.	6.68 [0.62]	6.48, 6.87	6.20 [1.20]	5.82, 6.58	0.70	0.403	1.08 [0.91, 1.28]	1.93
The interviewer was friendly.	6.50 [0.93]	6.20, 6.80	6.28 [1.30]	5.86, 6.69	0.15	0.696	1.04 [0.87, 1.23]	0.33
The interview was difficult.	2.35 [1.49]	1.87, 2.83	2.40 [1.45]	1.94, 2.86	0.00	0.957	0.99 [0.75, 1.32]	0.23
The interview involved a lot of cognitive load (mental effort).	3.70 [1.98]	3.07, 4.33	3.35 [2.03]	2.70, 4.00	1.46	0.228	1.16 [0.91, 1.48]	0.30
I reported a lot of details in the interview.	5.65 [1.19]	5.27, 6.03	4.88 [1.51]	4.39, 5.36	2.29	0.131	1.16 [0.96, 1.41]	3.73
I reported details that were important in the interview.	6.18 [0.93]	5.88, 6.47	4.63 [1.28]	4.22, 5.03	8.90	0.003	1.34 [1.10, 1.62]	428,504.51
I reported details that were NOT important in the interview.	4.18 [1.75]	3.61, 4.74	4.15 [1.72]	3.60, 4.70	0.01	0.941	1.01 [0.81, 1.25]	0.23
I expected I would be asked more questions in the interview.	5.08 [1.86]	4.48, 5.67	5.38 [1.48]	4.90, 5.85	0.43	0.511	0.94 [0.77, 1.14]	0.31
I expected I would be asked fewer questions in the interview.	3.05 [1.84]	2.46, 3.64	2.40 [1.46]	1.93, 2.87	3.86	0.050	1.32 [1.00, 1.76]	0.87
I am familiar with the store I reported visiting.	5.45 [1.88]	4.85, 6.05	5.93 [1.83]	5.34, 6.51	0.81	0.367	0.92 [0.76, 1.10]	0.41
I am familiar with the area(s)/street(s) I reported walking through.	6.75 [0.63]	6.55, 6.95	5.90 [1.81]	5.32, 6.48	2.29	0.131	1.14 [0.96, 1.36]	6.50

**Table 2:** Descriptive and inferential statistics for the 7-point scale ratings in the post-interview questionnaire as a function of Interview.

Questionnaire Item	Segmented		Structured		$\chi^{2}$ (1)	*p*	exp(B) [95% CI]	BF_10_
	M [SD]	95% CI	M [SD]	95% CI				
I was truthful during the interview.	4.43 [2.60]	3.59, 5.26	4.30 [2.77]	3.42, 5.18	0.46	0.500	1.09 [0.84, 1.42]	0.24
I was motivated to be believed by the interviewer.	6.08 [1.58]	5.57, 6.58	5.83 [1.68]	5.29, 6.36	0.24	0.622	1.05 [0.87, 1.25]	0.29
The interviewer believed me.	4.97 [1.17]	4.60, 5.35	4.90 [1.43]	4.44, 5.36	0.07	0.786	1.03 [0.84, 1.25]	0.24
The interviewer was listening carefully to me.	6.38 [1.13]	6.02, 6.73	6.50 [0.82]	6.24, 6.76	0.05	0.827	0.98 [0.83, 1.17]	0.27
The interviewer was friendly.	6.15 [1.33]	5.72, 6.58	6.63 [0.84]	6.36, 6.89	0.70	0.403	0.93 [0.78, 1.10]	1.11
The interview was difficult.	2.53 [1.36]	2.09, 2.96	2.23 [1.56]	1.73, 2.72	0.74	0.390	1.13 [0.85, 1.51]	0.33
The interview involved a lot of cognitive load (mental effort).	4.20 [1.80]	3.62, 4.78	2.85 [1.98]	2.22, 3.48	11.16	<0.001	1.50 [1.18, 1.91]	16.50
I reported a lot of details in the interview.	5.65 [1.17]	5.28, 6.02	4.88 [1.52]	4.39, 5.36	2.29	0.131	1.16 [0.96, 1.41]	3.73
I reported details that were important in the interview.	5.33 [1.25]	4.93, 5.72	5.48 [1.47]	5.01, 5.94	0.06	0.813	0.98 [0.81, 1.18]	0.26
I reported details that were NOT important in the interview.	4.08 [1.64]	3.55, 4.60	4.25 [1.82]	3.67, 4.83	0.15	0.699	0.96 [0.77, 1.19]	0.25
I expected I would be asked more questions in the interview.	4.40 [1.74]	3.84, 4.96	6.05 [1.13]	5.69, 6.41	10.55	0.001	0.73 [0.60, 0.88]	5087.00
I expected I would be asked fewer questions in the interview.	3.50 [1.62]	2.98, 4.02	1.95 [1.38]	1.51, 2.39	18.63	<0.001	1.83 [1.39, 2.43]	1184.94
I am familiar with the store I reported visiting.	5.80 [1.88]	5.20, 6.40	5.58 [1.85]	4.98, 6.17	0.20	0.657	1.04 [0.87, 1.25]	0.26
I am familiar with the area/street(s) I reported walking through.	6.38 [1.39]	5.93, 6.82	6.28 [1.45]	5.81, 6.74	0.03	0.859	1.02 [0.85, 1.21]	0.24

**Table 3:** Frequency of self-reported strategies by truth tellers and lie tellers in the post-interview questionnaire.

Strategy	Truth Tellers	Lie Tellers
Used an embedded lie	0	22
Reported person, location, action, temporal, and/or object details	15	17
Provided a detailed account	16	4
Provided an honest account	14	0
Reported on the surroundings	11	7
Provided a simple account	1	6
Monitored non-verbal behaviour	5	6
Tried to appear spontaneous/unrehearsed	5	5
Provided insignificant details	5	4
Provided a believable account	4	4
Acted in a friendly manner	4	0
Provided adequate details	3	3
Demonstrated confidence	3	2
Provided significant details	3	0

**Table 4:** Descriptive and inferential statistics for core and peripheral (PLATO) details and complications as a function of Veracity.

Detail Type	Truth Tellers		Lie Tellers		$\chi^{2}$ (1)	*p*	exp(B) [95% CI]	BF_10_
	M [SD]	95% CI	M [SD]	95% CI				
Core details	44.25 [23.14]	36.85, 51.65	36.93 [25.09]	28.90, 44.95	3.14	0.076	1.20 [0.98, 1.46]	0.52
Peripheral details	29.83 [27.57]	21.01, 38.64	15.90 [23.25]	8.47, 23.33	3.95	0.047	1.91 [1.01, 3.61]	2.95
Core complications	3.33 [2.48]	2.53, 4.12	1.13 [1.11]	0.77, 1.48	30.71	<0.001	2.83 [1.94, 4.20]	6642.64
Peripheral complications	2.38 [2.91]	1.44, 3.31	0.55 [1.18]	0.17, 0.93	16.56	<0.001	4.99 [2.27, 11.67]	61.41

**Table 5:** Descriptive and inferential statistics for core and peripheral (PLATO) details and complications as a function of Interview.

Detail Type	Segmented		Structured		$\chi^{2}$ (1)	*p*	exp(B) [95% CI]	BF_10_
	M [SD]	95% CI	M [SD]	95% CI				
Core details	55.35 [22.05]	48.30, 62.40	25.83 [16.10]	20.68, 30.97	55.06	<0.001	2.14 [1.76, 2.62]	5.38 × 10^6^
Peripheral details	32.28 [31.09]	22.33, 42.22	13.45 [15.86]	8.38, 18.52	7.37	0.007	2.43 [1.29, 4.60]	29.63
Core complications	2.98 [2.37]	2.22, 3.73	1.48 [1.77]	0.91, 2.04	10.81	0.001	1.89 [1.29, 2.80]	17.33
Peripheral complications	2.18 [2.70]	1.31, 3.04	0.75 [1.81]	0.17, 1.33	9.68	0.002	3.46 [1.57, 8.07]	6.05

**Table 6:** Simple effects for core and peripheral (PLATO) details and complications as a function of Veracity and Interview.

Detail Type		Truth Tellers		Lie Tellers		$\chi^{2}$ (1)	*p*	exp(B) [95% CI]	BF_10_
		M [SD]	95% CI	M [SD]	95% CI				
Segmented									
	Core details	60.35 [20.26]	50.87, 69.83	50.35 [23.12]	39.53, 61.17	1.66	0.198	1.20 [0.91, 1.58]	0.71
	Peripheral details	41.75 [30.04]	27.69, 55.81	22.80 [29.88]	8.82, 36.78	1.78	0.182	1.83 [0.75, 4.45]	1.45
	Core complications	4.55 [2.24]	3.50, 5.60	1.40 [1.14]	0.87, 1.93	23.45	<0.001	3.25 [2.02, 5.24]	6912.35
	Peripheral complications	3.45 [3.03]	2.03, 4.87	0.90 [1.52]	0.19, 1.61	7.77	0.005	3.83 [1.49, 9.86]	19.46
Structured									
	Core details	28.15 [11.94]	22.56, 33.74	23.50 [19.44]	14.40, 32.60	1.49	0.222	1.20 [0.90, 1.60]	0.43
	Peripheral details	17.90 [18.92]	9.04, 26.76	9.00 [10.79]	3.95, 14.05	2.25	0.134	1.99 [0.81, 4.89]	1.13
	Core complications	2.10 [2.13]	1.11, 3.09	0.85 [1.04]	0.36, 1.34	8.60	0.003	2.47 [1.35, 4.52]	2.62
	Peripheral complications	1.30 [2.41]	0.17, 2.43	0.20 [0.52]	−0.04, 0.44	7.77	0.005	6.50 [1.74, 24.23]	1.44

**Table 7:** Descriptive statistics for core and peripheral (PLATO) details in the free recall and follow-up responses as a function of Interview.

Detail Type		Segmented		Structured	
		M [SD]	95% CI	M [SD]	95% CI
Free recall					
	Core details	11.35 [7.54]	8.94, 13.76	12.80 [8.87]	9.96, 15.64
	Peripheral details	8.48 [8.26]	5.83, 11.12	6.23 [6.72]	4.07, 8.38
Follow-up responses					
	New core details	44.00 [19.62]	37.73, 50.27	13.03 [9.66]	9.93, 16.12
	New peripheral details	23.80 [24.59]	15.94, 31.66	7.23 [10.31]	3.93, 10.52

## Data Availability

The original data presented in the study are openly available at https://osf.io/kwzt7/.

## Supplementary Materials

The following supporting information can be downloaded at: https://www.mdpi.com/article/10.3390/bs15091163/s1, File S1: Interview protocol. File S2: Interview activities.

## References

[B1-behavsci-15-01163] Alison L., Alison E., Noone G., Elntib S., Waring S., Christiansen P. (2014). Whatever you say, say nothing: Individual differences in counter interrogation tactics amongst a field sample of right wing, AQ inspired and paramilitary terrorists. Personality and Individual Differences.

[B2-behavsci-15-01163] Bond C. F., DePaulo B. M. (2006). Accuracy of deception judgments. Personality and Social Psychology Review.

[B3-behavsci-15-01163] Brandon S. E., Wells S., Seale C. (2018). Science-based interviewing: Information elicitation. Journal of Investigative Psychology and Offender Profiling.

[B4-behavsci-15-01163] Caso A., Gabbert F., Dando C. J. (2024). Eyewitness confidence in the interviewing context: Understanding the impact of question type and order. Applied Cognitive Psychology.

[B5-behavsci-15-01163] Chan S., Bull R. (2014). The effect of co-offender planning on verbal deception. Psychiatry, Psychology and Law.

[B6-behavsci-15-01163] Collins A. M., Loftus E. F. (1975). A spreading-activation theory of semantic processing. Psychological Review.

[B7-behavsci-15-01163] Colwell K., Hiscock-Anisman C., Fede J., Cooper B., Griesel D., Ternes M. (2013). Assessment criteria indicative of deception: An example of the new paradigm of differential recall enhancement. Applied issues in investigative interviewing, eyewitness memory, and credibility assessment.

[B8-behavsci-15-01163] Danby M. C., Sharman S. J. (2023). Open-ended initial invitations are particularly helpful in eliciting forensically relevant information from child witnesses. Child Abuse & Neglect.

[B9-behavsci-15-01163] Dando C., Wilcock R., Milne R. (2009). The Cognitive Interview: The efficacy of a modified mental reinstatement of context procedure for frontline police investigators. Applied Cognitive Psychology.

[B10-behavsci-15-01163] Davis M. R., McMahon M., Greenwood K. M. (2005). The efficacy of mnemonic components of the cognitive interview: Towards a shortened variant for time-critical investigations. Applied Cognitive Psychology.

[B11-behavsci-15-01163] Deeb H., Granhag P. A., Vrij A., Strömwall L. A., Hope L., Mann S. (2018). Visuospatial counter-interrogation strategies by liars familiar with the alibi setting. Applied Cognitive Psychology.

[B12-behavsci-15-01163] Deeb H., Vrij A., Leal S., Fallon M., Mann S., Luther K., Granhag P. A. (2022). Mapping details to elicit information and cues to deceit: The effects of map richness. The European Journal of Psychology Applied to Legal Context.

[B13-behavsci-15-01163] Denne E., Brubacher S., Simpson K., Adams D., Dargue N., Powell M. (2024). Examining autistic and non-autistic children’s productivity in response to subtypes of open-ended prompts. International Journal on Child Maltreatment: Research, Policy and Practice.

[B14-behavsci-15-01163] DePaulo B. M., Lindsay J. J., Malone B. E., Muhlenbruck L., Charlton K., Cooper H. (2003). Cues to deception. Psychological Bulletin.

[B15-behavsci-15-01163] Evans J. R., Michael S. W., Meissner C. A., Brandon S. E. (2013). Validating a new assessment method for deception detection: Introducing a Psychologically Based Credibility Assessment Tool. Journal of Applied Research in Memory and Cognition.

[B16-behavsci-15-01163] Ewens S., Vrij A., Mann S., Leal S. (2015). Using the reverse order technique with non-native speakers or through an interpreter. Applied Cognitive Psychology.

[B17-behavsci-15-01163] Fisher R. P. (1995). Interviewing victims and witnesses of crime. Psychology, Public Policy, and Law.

[B18-behavsci-15-01163] Fisher R. P., Geiselman R. E. (1992). Memory enhancing techniques for investigative interviewing: The Cognitive Interview.

[B19-behavsci-15-01163] Fisher R. P., Schreiber Compo N., Rivard J., Hirn D., Lindsay D. S., Perfect T. J. (2014). Interviewing witnesses. The SAGE handbook of applied memory.

[B20-behavsci-15-01163] Gabbert F., Hope L., Luther K., Wright G., Ng M., Oxburgh G. (2021). Exploring the use of rapport in professional information-gathering contexts by systematically mapping the evidence base. Applied Cognitive Psychology.

[B21-behavsci-15-01163] Geiselman R. E. (2012). The cognitive interview for suspects (CIS). American Journal of Forensic Psychology.

[B22-behavsci-15-01163] Geiselman R. E., Fisher R. P. (1985). Interviewing victims and witnesses of crime.

[B23-behavsci-15-01163] Goldsmith M., Koriat A., Weinberg-Eliezer A. (2002). Strategic regulation of grain size memory reporting. Journal of Experimental Psychology: General.

[B24-behavsci-15-01163] Granhag P. A., Hartwig M. (2008). A new theoretical perspective on deception detection: On the psychology of instrumental mind-reading. Psychology, Crime & Law.

[B25-behavsci-15-01163] Granhag P. A., Hartwig M., Granhag P. A., Vrij A., Verschuere B. (2015). The strategic use of evidence technique: A conceptual overview. Detecting deception: Current challenges and cognitive approaches.

[B26-behavsci-15-01163] Granhag P. A., Strömwall L. A., Willén R., Hartwig M. (2013). Eliciting cues to deception by tactical disclosure of evidence: The first test of the Evidence Framing Matrix. Legal and Criminological Psychology.

[B27-behavsci-15-01163] Griffiths A., Milne R., Williamson T. A. (2006). Will it all end in tiers? Police interviews with suspects in Britain. Investigative Interviewing: Rights, research, regulation.

[B28-behavsci-15-01163] Hallgren K. A. (2012). Computing inter-rater reliability for observational data: An overview and tutorial. Tutorials in Quantitative Methods for Psychology.

[B29-behavsci-15-01163] Hartwig M., Granhag P. A., Stromwall L., Wolf A. G., Vrij A., Hjelmsäter E. R. A. (2011). Detecting deception in suspects: Verbal cues as a function of interview strategy. Psychology, Crime & Law.

[B30-behavsci-15-01163] Hartwig M., Granhag P. A., Strömwall L. A. (2007). Guilty and innocent suspects’ strategies during police interrogations. Psychology, Crime & Law.

[B31-behavsci-15-01163] Herlihy J., Jobson L., Turner S. (2012). Just tell us what happened to you: Autobiographical memory and seeking asylum. Applied Cognitive Psychology.

[B32-behavsci-15-01163] Hines A., CoIweII K., Hiscock-Anisman C., Garrett E., Ansarra R., Montalvo L. (2010). Impression management strategies of deceivers and honest reporters in an investigative interview. European Journal of Psychology Applied to Legal Context.

[B33-behavsci-15-01163] Hope L., Mullis R., Gabbert F. (2013). Who? What? When? Using a timeline technique to facilitate recall of a complex event. Journal of Applied Research in Memory and Cognition.

[B34-behavsci-15-01163] Jarosz A. F., Wiley J. (2014). What are the odds? A practical guide to computing and reporting Bayes factors. The Journal of Problem Solving.

[B35-behavsci-15-01163] Johnson M. K., Raye C. L. (1981). Reality monitoring. Psychological Review.

[B36-behavsci-15-01163] Kontogianni F., Hope L., Taylor P. J., Vrij A., Gabbert F. (2020). Tell me more about this…: An examination of the efficacy of follow-up open questions following an initial account. Applied Cognitive Psychology.

[B37-behavsci-15-01163] Koriat A., Goldsmith M. (1994). Memory in naturalistic and laboratory contexts: Distinguishing the accuracy-oriented and quantity-oriented approaches to memory assessment. Journal of Experimental Psychology: General.

[B38-behavsci-15-01163] Lamb M. E., Orbach Y., Sternberg K. J., Aldridge J. A. N., Pearson S., Stewart H. L., Esplin P. W., Bowler L. (2009). Use of a structured investigative protocol enhances the quality of investigative interviews with alleged victims of child sexual abuse in Britain. Applied Cognitive Psychology: The Official Journal of the Society for Applied Research in Memory and Cognition.

[B39-behavsci-15-01163] Leal S., Vrij A., Deeb H., Jupe L. (2018). Using the model statement to elicit verbal differences between truth tellers and liars: The benefit of examining core and peripheral details. Journal of Applied Research in Memory and Cognition.

[B40-behavsci-15-01163] Leins D. A., Fisher R. P., Pludwinsky L., Robertson B., Mueller D. H. (2014). Interview protocols to facilitate human intelligence sources’ recollections of meetings. Applied Cognitive Psychology.

[B41-behavsci-15-01163] Leins D. A., Fisher R. P., Ross S. J. (2013). Exploring liars’ strategies for creating deceptive reports. Legal and Criminological Psychology.

[B42-behavsci-15-01163] Mac Giolla E., Granhag P. A., Vernham Z. (2017). Drawing-based deception detection techniques: A state-of-the-art review. Crime Psychology Review.

[B43-behavsci-15-01163] Maier B. G., Niehaus S., Wachholz S., Volbert R. (2018). The strategic meaning of CBCA criteria from the perspective of deceivers. Frontiers in Psychology.

[B44-behavsci-15-01163] Meissner C. A., Redlich A. D., Michael S. W., Evans J. R., Camilletti C. R., Bhatt S., Brandon S. (2014). Accusatorial and information-gathering interrogation methods and their effects on true and false confessions: A meta-analytic review. Journal of Experimental Criminology.

[B45-behavsci-15-01163] Memon A., Meissner C. A., Fraser J. (2010). The Cognitive Interview: A meta-analytic review and study space analysis of the past 25 years. Psychology, Public Policy, and Law.

[B46-behavsci-15-01163] Memon A., Wark L., Holley A., Bull R., Köhnken G. (1997). Eyewitness performance in cognitive and structured interviews. Memory.

[B47-behavsci-15-01163] Murre J. M., Dros J. (2015). Replication and analysis of Ebbinghaus’ forgetting curve. PLoS ONE.

[B48-behavsci-15-01163] Nahari G., Ashkenazi T., Fisher R. P., Granhag P., Hershkowitz I., Masip J., Meijer E. H., Nisin Z., Sarid N., Taylor P. J., Verschuere B., Vrij A. (2019). ‘Language of lies’: Urgent issues and prospects in verbal lie detection research. Legal and Criminological Psychology.

[B49-behavsci-15-01163] Nunan J., Stanier I., Milne R., Shawyer A., Walsh D. (2020). Source Handler telephone interactions with covert human intelligence sources: An exploration of question types and intelligence yield. Applied Cognitive Psychology.

[B50-behavsci-15-01163] Oxburgh G. E., Myklebust T., Grant T. (2010). The question of question types in police interviews: A review of the literature from a psychological and linguistic perspective. International Journal of Speech, Language & The Law.

[B51-behavsci-15-01163] Paulo R. M., Albuquerque P. B., Vitorino F., Bull R. (2017). Enhancing the cognitive interview with an alternative procedure to witness-compatible questioning: Category clustering recall. Psychology, Crime & Law.

[B52-behavsci-15-01163] Powell M. B., Snow P. C. (2007). Guide to questioning children during the free-narrative phase of an investigative interview. Australian Psychologist.

[B53-behavsci-15-01163] Richardson G., Gudjonsson G. H., Kelly T. P. (1995). Interrogative suggestibility in an adolescent forensic population. Journal of Adolescence.

[B54-behavsci-15-01163] Russano M. B., Meissner C. A., Atkinson D. J., Brandon S. E., Wells S., Kleinman S. M., Ray D. G., Jones M. S. (2024). Evaluating the effectiveness of a 5-day training on science-based methods of interrogation with U.S. federal, state, and local law enforcement investigators. Psychology, Public Policy, and Law.

[B55-behavsci-15-01163] Semmler C., Dunn J., Mickes L., Wixted J. T. (2018). The role of estimator variables in eyewitness identification. Journal of Experimental Psychology: Applied.

[B56-behavsci-15-01163] Soufan A. H. (2011). The black banners: The inside story of 9/11 and the war against Al Qaeda.

[B57-behavsci-15-01163] Strömwall L. A., Willén R. M. (2011). Inside criminal minds: Offenders’ strategies when lying. Journal of Investigative Psychology and Offender Profiling.

[B58-behavsci-15-01163] Tekin S., Granhag P. A., Strömwall L., Giolla E. M., Vrij A., Hartwig M. (2015). Interviewing strategically to elicit admissions from guilty suspects. Law and Human Behavior.

[B59-behavsci-15-01163] U.K. Ministry of Justice (2022). Achieving best evidence in criminal proceedings: Guidance on interviewing victims and witnesses, and guidance on using special measures.

[B60-behavsci-15-01163] van Beek M. L., Bull R., Mijalkovic S. (2022). A free account or not? Its effect upon information yield in strategic interviews with suspects. Journal of Investigative Psychology and Offender Profiling.

[B61-behavsci-15-01163] Verschuere B., Bogaard G., Meijer E. (2021). Discriminating deceptive from truthful statements using the verifiability approach: A meta-analysis. Applied Cognitive Psychology.

[B65-behavsci-15-01163] Vrij A., Fisher R. P., Leal S. (2023). How researchers can make verbal lie detection more attractive for practitioners. Psychiatry, Psychology and Law.

[B63-behavsci-15-01163] Vrij A., Leal S., Fisher R. P., Mann S., Jo E., Shaboltas A., Khaleeva M., Granskaya J., Houston K. (2019). Eliciting information and cues to deceit through sketching in interpreter-based interviews. Applied Cognitive Psychology.

[B62-behavsci-15-01163] Vrij A., Leal S., Granhag P. A., Mann S., Fisher R. P., Hillman J., Sperry K. (2009). Outsmarting the liars: The benefit of asking unanticipated questions. Law and Human Behavior.

[B64-behavsci-15-01163] Vrij A., Palena N., Leal S., Caso L. (2021). The relationship between complications, common knowledge details and self-handicapping strategies and veracity: A meta-analysis. The European Journal of Psychology Applied to Legal Context.

[B66-behavsci-15-01163] Walsh D., Oxburgh G., Redlich A., Myklebust T. (2016). International developments and practices in investigative interviewing and interrogation: Volume 1: Victims and witnesses.

[B67-behavsci-15-01163] Wells G. L., Memon A., Penrod S. D. (2006). Eyewitness evidence: Improving its probative value. Psychological Science in the Public Interest.

[B68-behavsci-15-01163] Winter B., Bürkner P. C. (2021). Poisson regression for linguists: A tutorial introduction to modelling count data with brms. Language and Linguistics Compass.

