# Supplementary material for: The Segmented Interview: Partitioning the Initial Free Recall Topics into Segments to Enhance Information Gathering and Lie Detection
Source: Behav Sci (Basel). 2025 Aug 26;15(9):1163. doi: 10.3390/bs15091163 (PMC12466768; doi:10.3390/bs15091163)
Supplement: Supplementary file 1 [file behavsci-15-01163-s001.zip › Supplementary material_interview protocol.pdf]

<u>Instructions to Interviewers</u>

<u>Prior to the Interview</u>

- *The experimenter will ask you to go to their room while waiting for the participant to finish the final activity.*
- *Once the participant is back in the experimenter's room, instruct them as follows:*

"An envelope with money disappeared from room 1.10, and everyone currently in the department will be interviewed. I will thus be interviewing you in a few minutes."

- *Leave the room and return back after three minutes to take the participant to the interview room.*
- *During those three minutes, the experimenter will send you the participant number and Interview condition (Structured Interview or Segmented Interview).*

<u>The Interview</u>

1. **<u>Free Recall – All Participants</u>**

Start recording

"This is participant no. _______

It came to my attention that an envelope with money went missing during the past half an hour or so. I am thus interviewing all people present within the department. Please tell me about your whereabouts over the past half an hour or so."

*** Participant responds ***

- *Never change the wording of the Free recall question.*
- *Throughout the whole interview:*
    - *Remain friendly and act as if it is 'the best story they ever heard' and never interrupt the participant.*
    - *Even if you feel you know if the participant is telling the truth or not, please do not show this.*

2. **<u>Follow-up Questions</u>**

"I will be asking you for more information to clarify what you previously mentioned."

- *Ask the follow-up questions of either The Structured Interview or The Segmented Interview.*

Participant responds to each question

"Thank you, that is the end of my questions. You can now return back to the experimenter."

Examples of Follow-up Questions for the Structured Interview[1]

Ask only one question about each activity that was reported in the initial free recall. The questions should be based on the activities mentioned in the free recall. The below are only examples for guidance.

1. You mentioned going to a room to watch a video. Tell me everything you can remember around that time.
2. You mentioned going to a store to look for an item. Tell me everything you can remember around that time.
3. You mentioned going to a lecturer's office to check if she was there. Tell me everything you can remember around that time.
4. You mentioned going to a lounge to drink. Tell me everything you can remember around that time.

Examples of Follow-up Questions for the Segmented Interview

Ask four open questions about each activity that was reported in the initial free recall. The questions should be based on the activities mentioned in the free recall. The below are only examples for guidance.

1. You mentioned going to a room to watch a video.
   a) Tell me everything you can remember on the way to the room.
   b) Tell me everything you can remember about what you did while in the room.
   c) Tell me everything you can remember about the room.
   d) Tell me everything you can remember on the way from the room.

[1] Interviewers were provided with more example follow-up questions for the Segmented Interview than for the Structured Interview because the former were more difficult to formulate.

2. You mentioned going to a store to look for an item.
    a) Tell me everything you can remember on the way to the store.
    b) Tell me everything you can remember about what you did while in the store.
    c) Tell me everything you can remember about the store.
    d) Tell me everything you can remember on the way from the store.

3. You mentioned going to a lecturer's office to check if she was there.
    a) Tell me everything you can remember on the way to the office.
    b) Tell me everything you can remember about what you did while next to the lecturer's office.
    c) Tell me everything you can remember about the lecturer's office.
    d) Tell me everything you can remember on the way from the lecturer's office.

4. You mentioned going to a lounge to drink.
    a) Tell me everything you can remember on the way to the lounge.
    b) Tell me everything you can remember about what you did while in the lounge.
    c) Tell me everything you can remember about the lounge.
    d) Tell me everything you can remember on the way from the lounge.

5. You mentioned meeting a friend (*unspecified location*).
    a) Tell me everything you can remember on the way before meeting your friend.
    b) Tell me everything you can remember about what you did while with your friend.
    c) Tell me everything you can remember about the area where you met your friend.
    d) Tell me everything you can remember on the way after meeting your friend.

6. You mentioned meeting a friend in Guildhall Square.
    a) Tell me everything you can remember on the way to Guildhall Square (*priority to location*).
    b) Tell me everything you can remember about what you did while in Guildhall Square.
    c) Tell me everything you can remember about Guildhall Square.
    d) Tell me everything you can remember on the way from Guildhall Square.

- Do not ask questions about activities relevant to **room 2.28/experimenter/experimenter's office/etc**.
- Use the **participant's own words** (from the free recall) for formulating the follow-up questions.
- Only ask **open, Tell questions**.

- **Use the question wording above** as much as possible to standardise the questions.
- You do not have to ask about **location/action** if not specified by the participant in the free recall.
- When dividing the Free recall, divide it in terms of **location** (e.g., room, shop, lounge, Square).
- The questions above can be asked if the participant mentions the following locations/activities:
  - Going to a: room; store; lecturer's office; lounge; Guildhall Square/meeting a friend.
- Ask other questions if any other location or activity is mentioned by the participant.
- **Never** ask questions about locations/activities **not mentioned** by the participant in the free recall.
- The **order of questions** should be based on how the information was remembered in the free recall.
  - That is, if the participant started by discussing the store visit then talked about the video, the first question(s) should be about the store followed by the question(s) about the video.
- It may help to use the provided excel file (Interviewer_Activities).
- For the **Structured Interview**:
  - Ask **only one question** for each activity.
  - Each Structured Interview question is to be **phrased** as follows: "You mentioned going to a (location) to (participant's action). Tell me everything you can remember around that time."
- For the **Segmented Interview**, ask a **maximum of four questions** for each activity**:**
  - For each topic, start with: "You mentioned going to a (location) to (participant's action)."
  - Two questions should be about what happened 'on the way to/from the activity's location': "Tell me everything you can remember on the way to (location)/from (location)".
  - One (action) question should be about what happened during the corresponding activity: "Tell me everything you can remember about what you did while (*Priority is given to location but if there is no location then you can ask about action*)."
  - One (spatial) question should be about the activity location: "Tell me everything you can remember about (location)."
